# Two new cave rnicolous genera of Julidae  (Diplopoda, Julida), with notes on the tribe Brachyiulini and on julid subanal hooks and anchors

**DOI:** 10.3897/zookeys.114.1490

**Published:** 2011-06-30

**Authors:** Nesrine Akkari, Pavel Stoev, Henrik Enghoff

**Affiliations:** 1 Natural History Museum of Denmark (Zoological Museum), University of Copenhagen, Universitetsparken 15, DK-2100 København Ø, Denmark; 2 National Museum of Natural History, 1, Tsar Osvoboditel Blvd, 1000 Sofia and Pensoft Publishers, 13a, Geo Milev Str., 1111 Sofia, Bulgaria

**Keywords:** Greece, Italy, cave, millipedes, new genera, new species, *Titanophyllum*, *Mammamia*

## Abstract

Two remarkable genera and species of the millipede family Julidae, *Titanophyllum spiliarum* **gen. n., sp. n.** and *Mammamia profuga* **gen. n. sp., n.**, are described from caves in Greece and Italy, respectively. The presence of a flagellum and the absence of a ‘pro-mesomerital forceps’ on the gonopods place them in the tribe Brachyiulini Verhoeff, 1909, an unnatural grouping based on plesiomorphic characters. Both are outstanding in being the only hitherto known blind julidans having such gonopodal features. A dichotomous key to the nine valid brachyiulinine genera based on peripheral and gonopodal characters is presented. Moreover, notes on subanal hooks and anchors in Julida are provided with hypotheses on their possible function.

## Introduction

We describe here two new genera and species of Julidae, collected from caves in Italy and Greece, which we tentatively assign to the poorly characterised tribe Brachyiulini. In addition to the taxonomic description we discuss the status of the tribe and provide a dichotomous key to its nine currently valid genera. Notes on the presence of subanal hooks and anchors in Julida are also given with hypotheses on their possible function.

## Material and methods

Specimens were collected in two caves in Italy and Greece and preserved in 70% ethanol. All measurements were made using a Leica Wild M10 microscope equipped with an ocular micrometer. Vertical body diameter was measured at midbody. Antennae, legs and gonopods were mounted in glycerin for temporary microscope preparations.

Microphotographs were obtained using a Leica digital camera M205A mounted on a stereomicroscope Leica DFC 420. Images were processed with a Leica Application Suite program and final stacking made with Helicon Focus 4.60.2 Pro software. SEM micrographs were obtained using a JEOL JSM-6335F scanning electron microscope. Drawings were made using a camera lucida mounted on a Leica DMRXE microscope. All pictures were later assembled for a final layout with Adobe Photoshop CS.

## Results

### Taxonomy

**Order Julida Brandt, 1833**

**Family Julidae Leach, 1814**

**Tribe Brachyiulini Verhoeff, 1909**

#### Mammamia gen. n.

urn:lsid:zoobank.org:act:16F56F2A-E20D-43FA-815D-2C1E242401E1

http://species-id.net/wiki/Mammamia

##### Diagnosis.

 Differs from all other genera of Brachyiulini by lacking ocelli and by having a distally expanded promerite and a slightly shorter posterior gonopod, the latter with a basally broad and distally slender mesomerital process mostly lodged in an opisthomerital furrow.

##### Etymology.

 The name derives from the Italian exclamation “Mamma mia” which came to our mind when we first saw this astonishing species. Gender feminine.

#### Mammamia profuga sp. n.

urn:lsid:zoobank.org:act:C319EC4E-FB69-4756-B1CE-609ACDE9F347

http://species-id.net/wiki/Mammamia_profuga

Figs 1 –8 

##### Material examined.

 Holotype: adult ♂ (broken into head and 6 body parts), Italy, Taranto, Grotta della Cava, iii.1964, P. Parenzan leg. (Natural History Museum of Denmark, Zoological Museum, University of Copenhagen – ZMUC).

##### Description of locality.

 The new species was collected in a cave in Taranto Province (south-eastern Italy). P. Parenzan (1984, in a letter to HE) wrote that the cave where the species have been collected was subsequently destroyed.

##### Etymology.

‘profuga’ in Latin means homeless; the name emphasizes the destroyed type locality of the species.

##### General description

 (all measurements in mm). Body uniformly pale yellowish, approximately 26 mm in length, vertical body diameter (height, H) 1.5, length/height ratio 17. Head: ocelli absent, frontal setae and setal sockets missing; gnathochilarium with 2 setae in apical parts of the stipites and with a seta on each lamella lingualis; 4 supralabral setae and a row of *ca* 12 labral setae; mandibular stipital lobes not expanded in males; antennal length *ca* 1.5×H. Body with 51 podous + 2 apodous rings and telson; striation moderately dense; setae apparently missing, probably broken off; legs yellowish, their length *ca* 1.5×H. Male first leg-pair reduced and hook-shaped. Telson blunt, preanal ring without projection, with at least 5 long setae; subanal scale with 2 long setae; anal valves pilose.

##### Gonopods.

**Figures 1−3.**
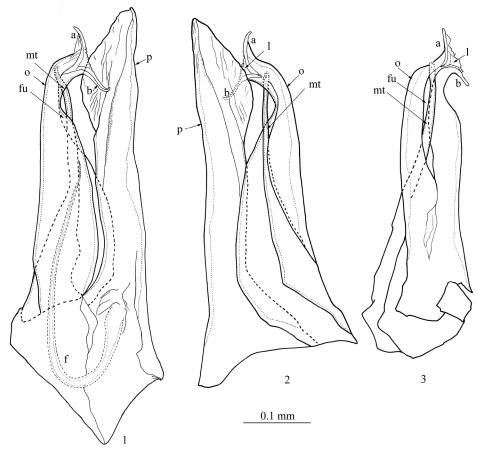
*Mammamia profuga* gen. n., sp. n., gonopods: **1** right gonopod, mesal view **2** right gonopod, lateral view **3** left posterior gonopod, anterior view. Abbreviations: *a*, *b*: opisthomerital processes *a* and *b*, *f*: flagellum, *fu*: furrow, *l*: lamella, *mt*: mesomerital process, *o*: opisthomerite, *p*: promerite.

**Figures 4−8.**
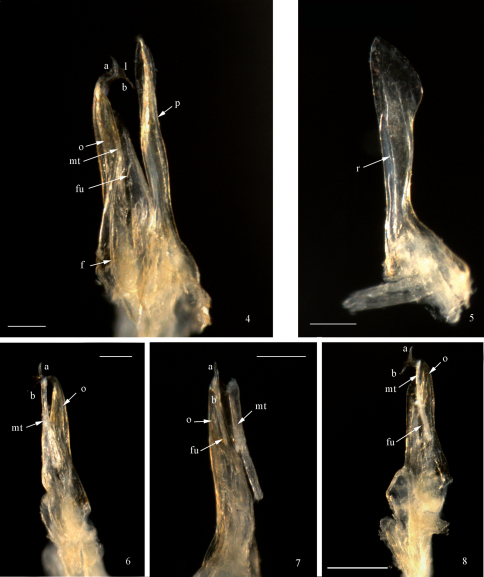
*Mammamia profuga* gen. n., sp. n., gonopods: **4** right gonopod, mesal view **5** left promerite, posterior view **6** left posterior gonopod, lateral view **7** left posterior gonopod, postero-lateral view **8** left posterior gonopod, posterior view. Abbreviations: *a*, *b*: opisthomerital processes *a* and *b*, *f*: flagellum, *fu*: furrow, *l*: lamella, *mt*: mesomerital process, *o*: opisthomerite, *p*: promerite, *r*: ridge. Scale bar: 0.1 mm.

Anterior gonopod (promerite, *p*) slightly longer than posterior gonopod (Figs 1, 2, 4); broad at base, abruptly narrowing at about 1/4 of its height, then gradually broadening distally to form a spatula-like process (Figs 1, 2, 5); apically blunt and mesally with a quite high ridge (*r*) (Fig. 5). Flagellum (*f*) (Figs 1, 4) moderately long, falcate, emerging from the promerite’s base, its tip reaching about 2/3 the height of the posterior gonopod. Posterior gonopod: Opisthomerite (*o*) (Figs 1−4, 6−8) broadest at base, gently tapering up to about 3/4 of its height, then abruptly narrowing and curving anterolaterad; mesally with a wide furrow (Figs 1, 2, *fu*) running along its length; apex resembling a fish-tail, with two processes (*a*, *b*) pointing in opposite directions (Figs 1−4, 6−8) connected by a thin, marginally serrated lamella (*l*) bearing several small spines on the surface (Figs 2−4). Mesomerital process (*mt*) (Figs 1−3, 4, 6−8) emerging from the anterior side of the opisthomerite, mostly lodged in the opisthomerital furrow, broad at base, narrowing at about midlength, thereafter becoming very slender and bent, apical margin gently serrated.

#### Titanophyllum gen. n.

urn:lsid:zoobank.org:act:C09ADA27-45ED-49E0-A2CB-1457D0CDF629

http://species-id.net/wiki/Titanophyllum

##### Diagnosis.

 Differs from all other genera of Brachyiulini by lacking ocelli and by having a rather simple, apically incised promerite devoid of any filamentous processes or apical appendages, and a simple unipartite posterior gonopod with a proximal lobe laterally and a subbasal fold and a groove mesally, the latter ending in a subapical opening.

##### Etymology.

The name combines the type locality, Titanospilia (the cave of Titans) and the suffix – *phyllum* – referring to the simple, leaf-shaped posterior gonopods. Gender neuter.

#### Titanophyllum spiliarum sp. n.

urn:lsid:zoobank.org:act:C4B8738C-470F-4F54-A4F4-B69741C20EF4

http://species-id.net/wiki/Titanophyllum_spiliarum

Figs 9 10 −14 

##### Material examined.

Holotype: adult ♂, Greece, Magnesia, Othris Mts., village of Kofi, Titanospilia (Cave of Titans), 13.VII.2003, P. Beron leg. (National Museum of Natural History Sofia − NMNHS); Paratypes: 4 ♂♂, 5 ♀♀, same locality, date and collector (ZMUC); 12 adult ♂♂, 11 adult ♀♀, 2 subadult ♂♂, 2 subadult ♀♀, same locality, date and collector (NMNHS).

##### Description of locality.

 Titanospilia is an approximately 100 m long vertical cave composed of a single voluminous hall. All material was collected at the bottom of the shaft (P. Beron, pers. comm.).

##### Etymology.

 The names means “of caves” in Greek and emphasizes the troglomorphic character of the species.

##### General description.

**Figure 9.**
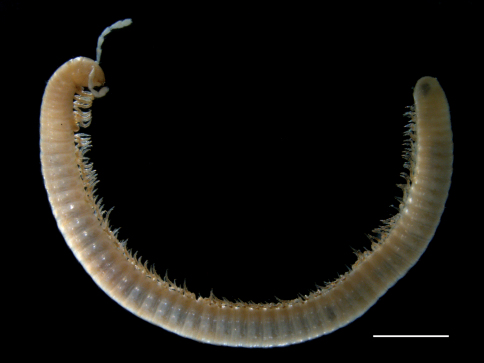
*Titanophyllum spiliarum* gen. n., sp. n., habitus. Scale bar: 2mm.

 (all measurements in mm). Body uniformly pale to yellowish, legs brownish, metazonites with a slightly darker posterior band; length: 17.3−33.5 mm, vertical body diameter (height, H) 1–1.2 (♂) and 1–1.4 (♀); length/height ratio 16 (♂). Head: ocelli absent (Fig. 9); frons with 2 setae; gnathochilarium with 3 setae in apical part of each stipites, and with a long seta on each lamella lingualis; 4 supralabral setae and a row of *ca* 8 labral setae; mandibular stipites not expanded; male first leg-pair reduced and hook-shaped; antennal length about 1.6×H. Body rings with more or less dense striation and a whorl of moderately dense long setae; 50–61 (♂) and 47–54 (♀) podous rings, 1–2 apodous rings + telson. Defense glands visible as dark spots opening on the suture. Length of legs *ca* 0.83×H. Preanal ring dorsally only slightly protruding beyond anal margin; subanal scale with a small hook pointing anteriad (Fig. 14); anal valves pilose, with long setae. Male 7th body ring with well developed ventral lobes.

##### Gonopods.

**Figures 10−13.**
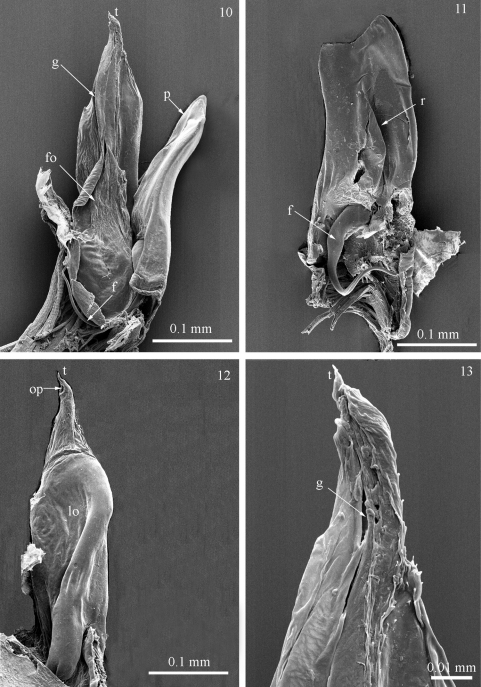
*Titanophyllum spiliarum* gen. n., sp. n., gonopods: **10** right gonopod, mesal view **11** left promerite, posterior view **12** right posterior gonopod, lateral view **13** close up of the tip of posterior gonopods, mesal view. Abbreviations: *f*: flagellum, *fo*: fold, *g*: groove, *lo*: lobe, *op*: opening, *p*: promerite, *r*: ridge, *t*: tip.

Gonopods protruding from the 7th body ring. Anterior gonopod (promerite, *p*) much shorter than posterior gonopod (Figs 10, 11), uniformly broad along its length, slightly expanded at midlength; apex incised, with a lower angular process mesally and a higher triangular one laterally (Fig. 11). Posterior side of promerite at middle with a quite high, semitriangular ridge (*r*) pointing postero-laterad (Fig. 11). Flagellum (*f*) (Figs 10, 11) moderately long, falcate, pointing ventrad, emerging from the base of ridge.

Posterior gonopod (Figs 12, 13) unipartite, long, broad at base and midlength, gradually tapering distally; with a proximal lobe (*lo*) laterally (Fig. 12) and a subbasal fold (*fo*) mesally (Fig. 10), the latter giving rise to a mesal groove (*g*) running proximal and distal (Figs 10, 13) and ending in a subapical opening (*op*) (Fig. 12). Gonopodal apex (*t*) with a pointed tip (Figs 12, 13).

## Discussion

The tribe Brachyiulini Verhoeff, 1909, is a grouping of julid genera based exclusively on plesiomorphic gonopod characters: presence of a flagellum and absence of a ‘pro-mesomerital forceps’ (Verhoeff 1926–1932, Enghoff 1987, Read 1990). The gonopods of some brachyiulinines do, however, have a process on the posterior gonopod which has been called ‘mesomerite’, ‘mesomerital process’, or ‘mesomere’ (see e.g., Verhoeff 1910, Schubart 1934, Golovatch et al. 2004). This process, unlike a ‘true’ mesomerite, is posterior rather than anterior as in most of the other julid tribes (Attems 1927) and does not form a forceps together with the promerite. Attems (1927) homologized this process with the posterior coxal process of *Cylindroiulus* and the paracoxal process of *Ommatoiulus* (as *Archiulus*). Whatever its name, this process cannot be regarded as a homologue of the ‘true’ mesomerite as seen in ‘higher julids’ or of the mesomerital processes as seen in Oncoiulini and Leucogeorginii (Enghoff 1987, Read 1990).

Hoffman (1980) listed 25 valid genera of Brachyiulini but noted that due to the general confusion that has applied to this tribe, he had “listed all names uncritically without regard to status, except in a few cases of very obvious synonymy”. Of the 25 genera, two (*Chromatoiulus* Verhoeff, 1894, and *Heteroiulus* Verhoeff, 1897) were moved to another tribe, Leucogeorgiini (Mauriès 1983, see also Enghoff 1987), and the status of *Campodes* Koch, 1847, is still unsettled (Hoffman 1999). Seven genera are currently considered as valid, viz., *Acropoditius* Strasser, 1980 – described as a subgenus of *Rhamphidoiulus*, elevated to full genus by Enghoff (2007); *Anaulaciulus* Pocock, 1895; *Balkanophoenix* Verhoeff, 1937; *Brachyiulus* Berlese, 1883; *Grusiniulus* Lohmander, 1936; *Megaphyllum* Verhoeff, 1894 and *Rhamphidoiulus* Attems, 1905. With about 17 subgenera (some of them considered synonyms) *Megaphyllum* is by far the largest but also the most problematical brachyiulinine genus. The last comprehensive study of this genus (as *Chromatoiulus*) was given by Attems (1927) and subsequently by (Strasser (1974, 1976). Golovatch et al. (2004) reviewed the subgenus *Persebrachyiulus* Golovatch, 1983 and commented on subgenus *Cyphobrachyiulus* Verhoeff, 1900, and Lazányi and Korsós (2011) revised the type species of *Megaphyllum*.

The assignment of *Titanophyllum* gen. n. and *Mammamia* gen. n. to Brachyiulini is based on purely typological considerations. They have gonopodal flagella and no mesomerites and therefore fall into the brachyiulinine ‘pigeonhole’. In the classical key to subfamilies/tribes of Julidae by Verhoeff (1926–32: 1647 ff), both genera easily run to couplet XI/XII (Heteroiulinae vs. Brachyiulinae) but because of their blindness do not make it all the way to Brachyiulinae.

Non-monophyly of Brachyiulini as currently defined is quite probable and is indeed suggested by recent molecular analyses of the phylogeny of the family Julidae (Enghoff et al. 2011). In the cladogram of these authors, based on partial sequences of the mitochondrial 16S rRNA (16S) and the nuclear 28S rRNA (28S) genes, and including 40 species and 22 genera of julids, two of the three included brachyiulinine genera, *Brachyiulus* and *Megaphyllum*, appear as sister genera but well apart from the third, *Anaulaciulus*. Contrastingly, the latter forms a clade with *Nepalmatoiulus* Mauriès, 1983, a morphologically distant genus having the characteristic pro-/mesomerital forceps. Both, however, share the trait of being the only julids occurring in South East Asia (Enghoff et al. 2011).

Even though all genera of Brachyiulini agree in the absence of a ‘pro-mesomerital’ forceps and the presence of a flagellum, the brachyiulinine gonopods exhibit great variation in the degree of complexity, size and position of the processes (see Table 1). The nine valid genera also vary in peripheral characters such as the presence/absence of ocelli, frontal setae and preanal hook, as well as the length of the preanal projection. The peripheral character that best characterises the new genera described here vis-a-vis the rest of the tribe is the lack of eyes.

**Table 1.** Characters matrix for the brachyiulinine genera. Ac – *Acropoditius*; An – *Anaulaciulus*; Ba – *Balkanophoenix*; Br – *Brachyiulus*; Gr – *Grusiniulus*; Me – *Megaphyllum*; Rh – *Rhamphidoiulus*; Tit – *Titanophyllum*; Mam – *Mammamia*

*Character*	*Ac*	*An*	*Ba*	*Br*	*Gr*	*Me*	*Rh*	*Tit*	*Mam*
Eyes	+	+	+	+	+	+	+	-	-
Frontal setae		+		+	+	+	+/-	+	-?
Subanal hook	-	-	-	-	-	-	-	+	-
Male mandibles with protruding lobe	+	-	-	+(all?)	+	+(all?)	+	-	-
Length of anterior gonopods compared to posterior gonopods	~as long	~half as long	~as long	~half as long	~half as long	~as long	~as long	¾ as long	longer
Anterior gonopods: flagelliferous lobe with very long, almost filamentous appendage	-	-	-	-	-	-	+	-	-
Anterior gonopods with a distal very long, almost filamentous appendage	+	-	-	-	-	-	-	-	-
Posterior gonopods simple, unipartite (S) vs. complex, bearing one or more processes distally (C)	S	C	C	C	C	C	S	S	C

### Key to the genera of Brachyiulini based on gonopods and peripheral characters

**Table:** 

1(4)	Ocelli absent	2
2(3)	Subanal scale with a hook; promerite broad, incised apically, much shorter than posterior gonopods	*Titanophyllum* gen. n.
3(2)	Subanal scale without a hook; promerite slenderer, distally expanded, slightly longer than posterior gonopods	*Mammamia* gen. n.
4(1)	Ocelli present	5
5(6)	Promerite with a long, falcate appendage apically	*Acropoditius*
6(5)	Promerite without such an appendage	7
7(8)	Promerite with a long, straight filamentous process at midlength; posterior gonopods simple	*Rhamphidoiulus*
8(7)	Promerite a filamentous process; posterior gonopods complex	9
9(15)	Promerite half as long as posterior gonopod	10
10(11)	Male mandibular stipes with expanded lobes (Southeast Asia)	*Anaulaciulus*
11(12)	Male mandibular stipes without a lobe (Europe, Caucasus)	13
13(14)	Promerite simple; posterior gonopods with an anterior ‘mesomerital’ process	*Brachyiulus*
14(13)	Promerite complex, with a well developed ridge and a basal cavity mesally, apex subconcave; posterior gonopods without anterior ‘mesomerital’ process	*Grusiniulus*
15(9)	Promerite nearly as long as posterior gonopod	16
16(17)	Male mandibular stipes without a lobe; metazonital striation absent; mesomerital process lying well apart from the main stem of the posterior gonopods, separated by a deep, broad concavity	*Balkanophoenix*
17(16)	Male mandibular stipes with a lobe; metazonital striation present; mesomerital process usually lying in close proximity to the main stem of posterior gonopods	*Megaphyllum*

### Hooks and anchors in julid millipedes

Apart from the male gonopods, julid millipedes are relatively uniform in structure. There are some non-gonopodal characters in males that exhibit a certain degree of variability, as do the female cyphopods, but when it comes to non-sexual morphology, the diversity is modest and mostly concerns such details as size, colour, presence/absence of eyes, presence/absence of frontal and metazonital setae, length of preanal projection, etc. There are a few julid species, however, which stand out by having some remarkable apomorphies. The first such species to be described was *Unciger foetidus* (C.L. Koch, 1838). The generic name *Unciger* Brandt, 1841, refers to its peculiar character: a stout forward-pointing hook on the subanal scale (Fig. 15). The hook occurs in both sexes. Waga (1839) gave a detailed description of the early postembryonic development of *Unciger foetidus* (under the name *Iulus unciger* Waga, 1839). He found that the subanal hook first makes its appearance in individuals with 15 pairs of legs, belonging to stadium III (cf. Enghoff et al. 1993). Two further species of the genus *Unciger* have been described, *Unciger transsilvanicus* (Verhoeff, 1899) (Fig. 15) and *Unciger kubanus* Lohmander, 1936, both having the characteristic subanal hook.

Strasser (1974) described another hook-bearing species: *Typhloiulus (?) uncinifer*, based on a juvenile collected on Cephalonia Island in Greece. As the question mark indicates, the assignment of this species to *Typhloiulus* is tentative – judged from the original description, the only indication in this direction is the lack of eyes. Ten years later, another julid with a hook on the subanal scale, *Syrioiulus andreevi* Mauriès, 1984 was described (Mauriès 1984), and now we add *Titanophyllum spiliarum* to the group possessing this peculiar character. We suspect that *Typhloiulus (?) uncinifer*, rather than belonging to the tribe Typhloiulini, may be related to *Typhloiulus spiliarum*. Even if this is not true, we now have hook-bearing species belonging to at least three tribes: Oncoiulini (*Unciger*), Pachyiulini (*Syrioiulus*), and Brachyiulini (*Titanophyllum*). Although the monophyly of Brachyiulini is questionable, there is no doubt that subanal hooks have arisen independently three times. A further argument in support of this statement is that all other species of *Syrioiulus* have no hooks. However, an undescribed *Syrioiulus* from Crete (Fig. 16) has a very prolonged subanal scale – perhaps a predecessor of the hook in *Syrioiulus andreevi* (Fig. 17).

Occurring in adults (both sexes) as well as juveniles, this hook has probably no function in courtship or copulation. One possibility could be that the hook is a protective device: when a julid rolls up into a spiral, as many julids do when disturbed, the hook might be inserted under one metazonital hind margin, ‘locking’ the spiral and making it more difficult for a would-be predator to uncoil. The only structure remotely similar to the julid hook we have been able to identify in other millipedes is the paxillus in certain Gomphodesmidae, order Polydesmida (Hoffman 2005). The paxillus is a forward-directed triangular process on the sternal part of the 15th body ring (Fig. 18). The topographical position of the paxillus would make a function similar to that suggested for the julid hook possible (and gomphodesmids do spiral when disturbed, HE pers. obs.). On the other hand, the paxillus occurs only in male gomphodesmids, so its function may be of a sexual rather than a generally defensive nature.

The julid hook may be a spiral-locking device, but there would be a different way obtaining the same effect if a structure on the dorsal side of the body rings could anchor itself to the leg coxae on the spiralled millipede. In fact there is a single species with such a structure, namely *Chersoiulus sphinx* Strasser, 1940, from north-eastern Italy, Slovenia and Croatia. In this species, each body ring carries a tiny mid-dorsal ‘anchor’ on the posterior margin of the metazonites (Fig. 19). The function of the anchors, which are missing in the only known congener, *Chersoiulus ciliatus* (Strasser, 1938), could very well be as suggested above for the subanal hooks. However, until somebody makes observations on behaviour of the hook- or anchor-bearing julids, the functional explanation given above remains entirely hypothetical.

**Figures 14−17.**
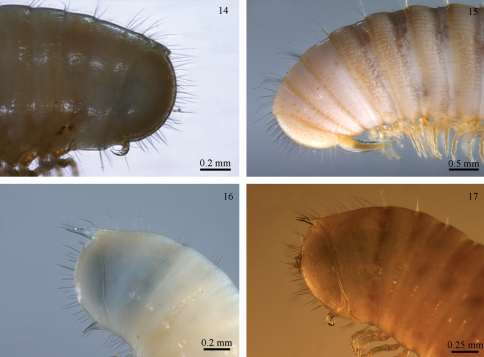
Hooks in julids: **14** *Titanophyllum spiliarum* **15** *Unciger transsilvanicus* (ZMUC) **16** *Syrioiulus* sp., Crete (ZMUC) **17** *Syrioiulus andreevi* (paratype, National Museum of Natural History, Sofia).

**Figures 18−19.**
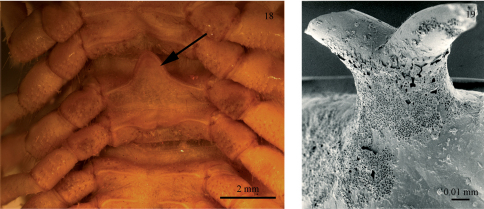
Anchors and sternal processes in millipedes: **18** Sternal process (indicated by an arrow) in *Astrodesmus laxus* (Gerstäcker, 1873) (Gomphodesmidae) (ZMUC) **19** Anchor in *Chersoiulus sphinx* (ZMUC).

## Supplementary Material

XML Treatment for Mammamia

XML Treatment for Mammamia profuga

XML Treatment for Titanophyllum

XML Treatment for Titanophyllum spiliarum
